# Observations on One of the Means by Which the Eye Has Been Supposed to Accomodate Itself to the Different Distances of Objects

**Published:** 1799-11

**Authors:** 


### *Observations on one of the Means by which the Eye has been supposed to accomodate itself to the different Distances of Objects.*

IN the late publication of Dr. MONRO on the brain, eye, and ear, after enumerating several means by which the eye accmomodates itself to the different distances of objects; he concludes by pointing out one of the means which had been overlooked by authors, and which we employ when we view minute objects near the eye. This, he observes, occurred to him from the circumstance, that when we view minute objects near the eye, we are sensible that we bring the upper and under eye-lids nearer to each other, and then, by a considerable exertion, contract the parts about the eye. The effect produced, to wit, the seeing more distinctly, he attributes to the orbicularis muscle of the eye, by its pressure, making the upper and under parts of the cornea somewhat flatter, and of course protruding the middle part of the cornea between the edges of the eyelids, so as to render it more convex. Hence the refractive power of the eye is increased, and the pencils of rays which would have united beyond, are made to unite upon the retina. Dr. MONRO conceives his opinion confirmed by three experiments, which he made for the purpose of discovering whether or not it was well founded.

In

In the 1st, he placed his eyes so near a small printed book, placed opposite a window, as that the letters appeared indistinct. He found he could neither read the book when he kept the eyelids in their ordinary state, nor when he opened them wide.

But in the 2d experiment, on acting with the orbicularis palpebrarum, so as to bring the eyelids within a quarter of an inch of each other, and making an exertion to read, he found he could see the letters and words distinctly.

And in the 3d, instead of employing the muscular contraction of the orbicularis, when he brought the eyelids within a quarter of an inch of each other, by means of his fingers, and then stretched the edges, so as to make a pressure upon the cornea, he found the letters appear distinct. He concludes by observing, that there can be no doubt of this action of the orbicularis producing the effect he attributes to it, as the distance of the eye from the object and the quantity of light were the same; as no part of the pupil was covered by the eyelids, so as to cut off the most diverging rays; and as the object appeared confused when the orbicularis was not contracted, but distinct on its contracting.

These experiments, however, do not appear to me to prove the point which Dr. Monro endeavours to establish. It is true, indeed, (as he remarks in the beginning of his Observations) that when we view small objects near the eye, we bring the upper and lower eyelids nearer each other, and by a forcible and rather painful exertion, contract the parts about the eyes. But these, I apprehend, we put in practice for a very different reason from that which Dr. Monro assigns.

When a small object is near the eye, some of the rays which proceed from every point of the object, and fall on every point of the cornea' are so oblique, as to be unable to be brought into a focus on the retina, by the refractive powers of the humours of the eye. Hence the object is confused: but if by means of a small hole in a card, interposed between the eye and the object, the more diverging rays are cut off, and those only which are less oblique, allowed to reach the eye; then the humours are able to effect the proper degree of refraction, and the object appears distinct. Upon this ground, I conceive an explanation of the doctor's experiments may be given: Some of the rays from the printed book fell too obliquely upon the cornea, to suffer the proper refraction. On bringing the eyelids, however, nearer each other, these superfluous rays were cut off, and the object appeared more distinct: but in Dr. Monro's experiments, it is said, that no part of the pupil was covered by by the eyelids, ſo as to intercept the diverging rays, which might tend to render the object confuſed. When the experiments are repeated, however, it will be found neceſſary for the eyelids to be conſiderably within a quarter of an inch of each other, to render the object more diſtinct, and that the diſtinctneſs is greater, if part of the pupil be covered by the eyelids.

But though the pupil ſhould not be at all covered by the eyelids, their being brought nearer each other than they uſually are, will have a tendency to intercept oblique rays; ſince the refractive power of the cornea and aqueous humour can make rays enter the pupil which fall upon the cornea, beyond that part of it which anſwers to the pupil in dimenſions.

With regard to the exertion we make in viewing a minute object, and the contraction we produce in the parts about the eye, which Dr. Monro conſiders as proofs of the forcible action of the orbicularis, they ſeem to depend,

1ſt. Upon the neceſſity there is that the optic axes ſhould unite in an object before we can have diſtinct ſingle viſion. And to produce this both eyes muſt be turned conſiderably inward, in viewing a minute object near the eye, which requires a painful exertion of the muſcles of the eye-ball, certainly referred by us to the internal parts.

2d. Upon the action of the muſcles of the eye-ball in increaſing the convexity of the cornea, by puſhing the aqueous humour forwards, which Dr. Hosack and Mr. Home have proved to be the uſual method in which the eye accommodates itſelf to different diſtances, and to which Dr. Monro imagines the action of the orbicularis is added.

3d. Upon the ſupport which the neighbouring muſcles when contracted give to the eye and its muſcles, the continued action of which ſoon becomes painful.

Beſides the circumſtance that a conſiderable degree of preſſure would be neceſſary to produce an alteration in the convexity of the cornea, ſuch as the orbicularis does not ſeem able to give upon ſo firm and thick a membrane as the cornea, and which we ſhould certainly feel if it were able to give it; I would obſerve, that ſimply bringing down the eye-lids with the fingers without ſtretching them, or without acting at all with the orbicularis, renders viſion more diſtinct; and here it is evident, that there can be no ſuch preſſure as that for which Dr. Monro contends.

To all these I would add, that myopes, or short sighted people, who have the refractive power of their eyes too considerable, almost always, in viewing an object, make the eye-lids approach more than the long-sighted, or those with vision in a healthy state; which should, according to the opinion of Dr. MONRO concerning the action of the orbicularis, increase the refractive power of their eyes, and consequently render their vision more imperfect, which by experience it is not found to do.

